# Symptom-based subtypes in obstructive sleep apnea: are we ready for global precision phenotyping?

**DOI:** 10.1093/annalsats/aaoag118

**Published:** 2026-05-06

**Authors:** Baran Balcan, Nesrin Ocal

**Affiliations:** Faculty of Medicine, Department of Pulmonary Medicine, Koç University, Istanbul, Turkey; Department of Pulmonary Medicine, University of Health Sciences, Gulhane Teaching and Education Hospital, Ankara, Turkey

## Introduction

Obstructive sleep apnea (OSA) is widely recognized as a heterogeneous disorder, yet clinical practice continues to rely heavily on simplified metrics such as the apnea–hypopnea index (AHI).^1^^,^^2^ Over the past decade, symptom-based phenotyping has emerged as a promising approach to better capture this heterogeneity and advance precision medicine.^1^ In this issue of the *Annals of the American Thoracic Society*, Yi and colleagues^3^ provide important new evidence supporting the generalizability of symptom-based subtypes across ethnically diverse populations, with a particular focus on Chinese patients with moderate-to-severe OSA.

Using data from the Sleep Apnea Global Interdisciplinary Consortium, an international, multicenter cohort, the authors analyzed 1863 patients from China and Western countries using latent class analysis.^3^ They demonstrate that 5 previously described symptom subtypes—minimal symptoms, disturbed sleep, excessively sleepy, moderately sleepy, and upper airway–dominant—are consistently reproducible across populations.^4^^,^^5^ These findings confirm earlier work and extend the evidence base by validating this framework in a large, clinic-based Chinese cohort.

## Moving beyond AHI: the case for symptom phenotyping

The limitations of AHI as a sole descriptor of OSA severity are well established. Patients with similar AHI values may exhibit markedly different symptom burdens, cardiovascular risks, and responses to therapy.^6^^,^^7^ Symptom-based subtyping offers a clinically intuitive and scalable framework to address this gap. Unlike physiologic phenotyping, which often requires complex measurements, symptom clusters can be derived from standardized questionnaires and implemented in routine care.

The present study reinforces that symptom phenotyping is not only reproducible but also globally applicable. The replication of the 5-subtype model across distinct populations suggests that these phenotypes capture core features of OSA expression rather than population-specific artifacts.^4^^,^^5^^,^^8^^,^^9^

## Ethnic differences: biology, culture, or both?

Although subtype structure was preserved, important differences in subtype prevalence were observed between Chinese and White patients. Chinese patients were more likely to belong to the “minimal symptoms” subtype despite having more severe OSA as measured by AHI. This apparent dissociation between objective severity and symptom burden has been described previously and raises important questions regarding symptom perception and reporting.^8^

Several mechanisms may underlie these differences. Anatomical factors, particularly craniofacial structure, may predispose Asian patients to more severe upper airway collapse at lower body mass indices.^10^^,^^11^ Cultural and behavioral factors may also influence symptom reporting, including differences in health awareness and healthcare access. Moreover, the global burden of OSA—particularly in Asian populations—underscores the importance of understanding these patterns.^12^

These findings suggest that symptom phenotypes reflect not only biological mechanisms but also sociocultural context, emphasizing the need for careful interpretation across populations.

## Clinical implications: toward precision sleep medicine

The reproducibility of symptom subtypes has important implications for clinical practice. Symptom-based classification may provide a practical tool for patient stratification and could inform individualized management strategies.^13^^,^^14^

Previous studies have shown that excessively sleepy patients derive the greatest symptomatic benefit from continuous positive airway pressure, whereas minimally symptomatic patients tend to demonstrate lower adherence.^6^^,^^7^ In addition, symptom subtypes have been associated with cardiovascular outcomes, further supporting their clinical relevance.^6^^,^^7^ The present study reinforces these observations by demonstrating that the excessively sleepy subtype is associated with greater disease severity.

However, symptom subtypes alone may not fully capture underlying pathophysiology. Integration with physiological traits and biomarkers will likely be required to achieve truly personalized treatment approaches.

## Stability and dynamics of symptom subtypes

An important consideration is that symptom subtypes are not static. Longitudinal studies indicate that a substantial proportion of patients transition between subtypes over time.^15^ This dynamic nature suggests that symptom phenotypes may reflect disease state rather than fixed disease categories.

This has important implications for clinical care. A single baseline classification may not be sufficient for long-term decision making, and repeated assessments or trajectory-based approaches may be necessary.

## Strengths and limitations

This study has several strengths, including its large, international sample and the use of standardized symptom assessments across centers.^3^ The inclusion of multiple Chinese cohorts allows for robust within- and between-population comparisons.

Limitations include the cross-sectional design, which precludes assessment of temporal stability, and reliance on self-reported symptoms, which may introduce reporting bias. In addition, the absence of standardized measures of psychological factors limits interpretation of symptom variability.

## The road ahead

Yi and colleagues^3^ provide compelling evidence supporting the global generalizability of symptom-based OSA subtypes. These findings represent an important step toward precision phenotyping in sleep medicine.

Future research should focus on linking symptom subtypes to treatment response and long-term outcomes, integrating symptom-based and physiological phenotyping, and identifying the determinants of symptom expression across populations.

Ultimately, the goal is not simply to classify patients but to improve outcomes. Symptom-based phenotyping offers a promising pathway—but its clinical value will depend on its ability to guide personalized, effective, and equitable care.

## Supplementary Material

aaoag118_Supplementary_Data
